# Bacitracin-Loaded Type I Collagen/Bacterial Cellulose Dressing for the Repair of Infected Wounds

**DOI:** 10.3390/jfb17080400

**Published:** 2026-08-13

**Authors:** Chengcheng Gong, Wenwen Jiang, Siyu Huai, Huiling Tong, Nan Tang, Xidong Wu, Haiyong Ao

**Affiliations:** 1 Department of General Medical, The First Affiliated Hospital, Jiangxi Medical College, Nanchang University, Nanchang 330013, China; 2 Nanchang Key Laboratory for Smart Biomaterials Regulation and Adaptation & School of Materials Science and Engineering, East China Jiaotong University, Nanchang 330013, China; 3 Department of Drug Safety Evaluation, Jiangxi Testing Center of Medical Device, Nanchang 330013, China; 4 Jiangxi Key Lab of Flexible Electronics & Flexible Electronics Innovation Institute, Jiangxi Science and Technology Normal University, Nanchang 330013, China

**Keywords:** infected wound, bacterial cellulose, bacitracin, antibacterial activities, cytocompatibility

## Abstract

In this study, bacitracin—a small-molecule antibacterial agent—was incorporated into the network structure of a type I collagen/bacterial cellulose (Col/BC) composite via in situ recombination and impregnation adsorption, leveraging the well-established drug-loading capacity and sustained-release characteristics of type I collagen. The obtained BA@Col/BC (BA@CBC) functional dressing features a well-defined nanoporous architecture, high porosity (83.9 ± 1.8%), rapid water absorption kinetics, substantial water absorption capacity (48.7 ± 2.4 g/g), and satisfactory moisture permeability (2984 ± 56 g·m^2^·day). Critically, the introduction of type I collagen not only effectively delays the release of bacitracin, but also significantly improves the cytocompatibility of the bacterial cellulose-based dressing. BA@CBC exhibits powerful antibacterial activities against *S. aureus* and *MRSA*, and can promote the proliferation of NIH3T3 and HUVEC, demonstrating excellent cytocompatibility. In vivo studies in a murine infected-wound model revealed that BA@CBC significantly accelerates wound closure compared to controls; histological evaluation further showed the most organized re-epithelialization, robust granulation tissue formation, and de novo hair follicle regeneration in the BA@CBC group. Fluorescence immunohistochemical analysis confirmed markedly reduced expression of pro-inflammatory cytokines IL-1β and TNF-α in BA@CBC-treated wounds, indicating effective suppression of excessive inflammation and consequent improvement of the wound microenvironment. Collectively, BA@CBC integrates favorable physicochemical properties, sustained antibacterial functionality, and superior cytocompatibility—positioning it as a promising candidate for clinical management of infected wounds.

## 1. Introduction

Open wounds on the skin caused by burns, scalds, or chronic injuries are highly susceptible to secondary infection; the complex wound microenvironment resulting from infection becomes a critical factor impeding wound healing [1,2,3]. As a naturally synthesized polysaccharide-based polymer, bacterial cellulose (BC) serves as a highly promising wound dressing substrate owing to its intrinsic high purity, exceptional tensile strength and dimensional stability, rapid fluid absorption capacity, high water vapor transmission rate (WVTR), and well-documented biocompatibility [4]. However, open skin wounds are highly susceptible to infection, yet BC itself lacks intrinsic antibacterial activity, rendering it inadequate for the management of infected wounds [5]. The incorporation of antibacterial agents represents an effective strategy to confer antimicrobial functionality upon dressings [6,7,8].

Bacitracin (BA) is a polypeptide antibiotic produced by *Bacillus subtilis* or *Bacillus licheniformis* [9]. Its chemical structure comprises a pentacyclic ring and a dihydrothiazole ring side chain linked via a lysine amide bond and an asparagine glycosyl group, a structural feature that confers resistance to proteolytic degradation. BA exhibits potent inhibitory activity against Gram-positive bacteria and demonstrates favorable antibacterial efficacy against *methicillin-resistant Staphylococcus aureus* (*MRSA*) [10]; its minimum inhibitory concentrations (MICs) against *S. aureus* and *MRSA* are 16 μg/mL and 32 μg/mL, respectively [11]. Furthermore, BA exerts a mild antibacterial effect with negligible cytotoxicity, making it suitable as an antimicrobial agent for skin dressings [12]. However, as a small-molecule drug, BA is prone to burst release when directly incorporated into BC.

To address the inherently weak binding affinity between BA and BC, our research group previously employed a polydopamine nano-coating-mediated covalent grafting strategy to immobilize BA onto the surface of BC nanofibers, thereby achieving effective and stable surface functionalization [13]. Nevertheless, since the dressing acts on the wound surface, the loaded drug must be released into the deeper tissue layers to exert its therapeutic effect. Drugs that are covalently grafted and immobilized are unable to reach or treat deep-seated wounds. Furthermore, the cytocompatibility of the BA-modified BC remains suboptimal and requires further enhancement. Therefore, an alternative strategy is required to achieve sustained release of BA.

Type I collagen (Col) exhibits excellent cytocompatibility, and promotes wound healing [14,15,16]; it is also commonly used as a drug carrier material with favorable sustained drug release properties [17,18,19]. We hypothesize that the incorporation of collagen into the BC network structure can reduce the release rate of BA, confer excellent cytocompatibility and wound-healing-promoting properties to BC-based dressings. In this study, Col was uniformly incorporated into the three-dimensional BC network via an in situ composite method, and BA was controllably loaded through an impregnation approach. The physicochemical and biological properties of the obtained dressings were systematically characterized, and their wound healing promotion and anti-infection care capabilities were thoroughly evaluated through animal model experiments using infected wounds.

## 2. Materials and Methods

### 2.1. Materials

Col from pigskin with a molecular weight of 3.0 × 10^5^ Da was obtained from Sichuan Minglet Biological Technology Co., Ltd. (Chengdu, China). BA was purchased from Shanghai Macklin Biochemical Technology Co., Ltd. (Shanghai, China). *Acetobacter xylinum X-2* was (supplied by the Institute of Microbiology, Chinese Academy of Sciences (Beijing, China).

### 2.2. Preparation of BA@Col/BC Dressing

BA@Col/BC dressing was prepared via a two-step process. First, Col/BC composites were prepared via in situ culture, as described in the literature [20]. Briefly, *Acetobacter xylinum X-2* was cultured in 1 mL BC culture medium containing 1 mg/mL Col for 3 days in a 24-well plate. The harvested Col/BC composites were purified by ultrasonic oscillation in 0.5% (*w*/*v*) NaOH solution for 0.5 h, followed by repeated washing with deionized water until neutral pH was achieved. After freeze-drying, the Col/BC aerogel was obtained and designated as CBC. Subsequently, the CBC aerogel was immersed in a 3 mg/mL BA solution and reacted for 6 h in a shaking incubator at 37 °C and 130 rpm. The resulting BA@Col/BC (noted as BA@CBC) was rinsed three times with deionized water and then freeze-dried.

For comparison, the BA@BC composite was prepared by immersing the BC aerogel in a 3 mg/mL BA solution for 6 h, following the same procedure described above.

### 2.3. Characterization

Surface topography of all specimens was assessed via scanning electron microscopy (SEM, SU8010, Hitachi, Hitachi, Japan). Porosity values for both bacterial cellulose (BC) and hydroxyalkyl chitosan/bacterial cellulose (HACC/BC) membranes were determined through liquid displacement method. Molecular functional groups present in the materials were identified by attenuated total reflectance Fourier-transform infrared spectroscopy (FTIR; DSQ II–Thermo Scientific Nicolet iS50, Thermo Fisher, Waltham, MA, USA). Phase composition and crystallinity of the films were evaluated using powder X-ray diffraction (XRD; D8 ADVANCE, Bruker, Germany).

### 2.4. Thermostability

Thermogravimetric behavior of the specimens was evaluated on a Pyris 1 thermogravimetric analyzer (PerkinElmer, Waltham, MA, USA), employing a heating ramp of 10 °C min^−1^ from 50 to 800 °C under a nitrogen atmosphere.

### 2.5. Water Absorbency and Water Vapor Transmission Rate

Air-dried specimens (initial weight, *W_0_*_0_) were immersed in deionized water at ambient temperature. At predetermined time points, each sample was carefully removed, blotted with absorbent filter paper to eliminate excess surface moisture, and immediately weighed (*W_t_*). The water uptake capacity (WAR, %) was then determined using the equation:

(1) $$\mathrm{WAR}\;(\%)\;=\;\frac{W_{t}-W_{0}}{W_{0}}\;\times \;100\%$$

Water vapor permeability of the membranes was determined according to ASTM E96-16 (desiccant method), following the procedure reported previously [21]. In brief, circular sample discs were mounted over the open mouth of 50 mL glass centrifuge tubes filled with anhydrous CaCl_2_, and the assemblies were placed in a controlled-environment chamber at 37 °C and 90% relative humidity. The mass gain of each tube was recorded daily over a 6-day period. The water vapor transmission rate (WVTR) was measured as follows:

(2) $$\mathrm{WVTR}\;(g\cdot m^{-2}\cdot {\mathrm{day}}^{-1})\;=\;\Delta m/\left(A\;\times \;t\right)$$

where Δm (g) is the total mass gain due to water vapor absorption, A (m^2^) refers to the exposed surface area available for vapor transport, and t (days) corresponds to the duration of the test.

### 2.6. The Release Kinetics of BA

Triplicate samples were immersed in 50 mL of phosphate-buffered saline (PBS) and agitated on an orbital shaker at 37 °C and 100 rpm. At scheduled intervals, 5 mL aliquots of the supernatant were withdrawn for UV–vis spectrophotometric quantification at 254 nm, and immediately replenished with an equal volume of fresh PBS to preserve sink conditions. The cumulative amount of BA released (*Q_n_*) was calculated using the equation:

(3) *Q_n_* = *C_n_* × *V*_0_ + (*C*_1_ + *C*_2_ + *C*_3_ + … + *C_n_*_−1_) × *V*

where *C_n_* represents the BA concentration measured in the supernatant at the nth time point, *V*_0_ is the initial volume of the release medium (50 mL), and *V* refers to the volume of each aliquot withdrawn for analysis (5 mL).

### 2.7. Antibacterial Properties

Antimicrobial activity of the BC-derived dressings was assessed via the agar well diffusion assay, in compliance with the Chinese national standard GB/T 20944, *Staphylococcus aureus* (*S. aureus*, ATCC 25923) and *Methicillin-Resistant Staphylococcus aureus* (*MRSA*, ATCC 43300) were used as the test strain. Bacterial suspension density was standardized to approximately 1 × 10^8^ CFU/mL using a McFarland 0.5 turbidity standard (Kont Bio-Chem Technology Co., Shenzhen, China). Specifically, 200 μL of the adjusted suspension was uniformly spread onto the surface of tryptic soy agar (TSA) plates. Sterile circular specimens (6 mm diameter) were then placed at the center of each plate, followed by incubation at 37 °C for 1, 4, and 7 d. Clear inhibition zones surrounding the samples were visually inspected and measured using digital calipers.

To gain deeper insight into the bactericidal mechanism of the BC-based dressings, individual specimens were incubated in 1 mL of bacterial suspension (1 × 10^6^ CFUs) within a 24-well microplate for 24 h at 37 °C under static conditions. The obtained bacterial/material samples were divided into two parts. The first portion was fixed with 2.5% (*v*/*v*) glutaraldehyde, subjected to graded ethanol dehydration (30–100%), critical-point dried, and subsequently imaged via SEM (SU8010, Hitachi, Hitachi, Japan). The second portion was stained using the LIVE/DEAD™ BacLight™ Bacterial Viability Kit (Thermo Fisher Scientific, Waltham, USA), and bacterial viability distribution was visualized by confocal laser scanning microscopy (CLSM; Leica TCS SP8, Leica Microsystems, Wetzlar, Germany).

### 2.8. Cytocompatibility

Cytocompatibility of the BA@CBC dressing was evaluated using two mammalian cell lines: murine fibroblast NIH-3T3 cells (purchased from the Cell Bank of the Chinese Academy of Sciences, Shanghai, China) and human umbilical vein endothelial cells (HUVECs; supplied by Beijing Yuhengfeng Technology Co., Ltd., Beijing, China). Both cell types were maintained in Dulbecco’s Modified Eagle Medium (DMEM; Thermo Fisher Scientific, Waltham, USA), supplemented with 10% heat-inactivated fetal bovine serum (FBS) and 1% penicillin–streptomycin solution, under standard culture conditions (37 °C, 5% CO_2_, humidified atmosphere). Sterilized dressings were placed at the bottom of 24-well plates, and 1 × 10^4^ cells per well were seeded directly onto the material surface. Cell viability was assessed after 1, 3, and 5 days of co-culture using both the Cell Counting Kit-8 (CCK-8) assay and fluorescein diacetate (FDA) live-cell staining.

Cell morphology on the various biomaterial surfaces was examined using SEM. NIH-3T3 or HUVECs were seeded onto BC-based composite specimens and cultured for 72 h. Following fixation with 2% (*v*/*v*) glutaraldehyde in phosphate buffer (0.1 M, pH 7.4), samples underwent sequential dehydration in increasing concentrations of ethanol (30–100%), followed by critical-point drying via HMDS substitution. Specimens were then sputter-coated with a thin layer of gold/palladium and imaged under high vacuum using an SEM (SU8010, Hitachi, Hitachi, Japan).

### 2.9. In Vivo Wound Healing

All animal procedures were approved by the Experimental Animal Ethics Committee of the Jiangxi Medical Device Testing Center (Approval No. PZ202001-1) and carried out in strict accordance with the ARRIVE guidelines 2.0. This study used a randomized, controlled, parallel-group design with the experimental unit as a single animal. Fifty male Kunming mice (10 weeks old, 30–35 g, specific pathogen-free) were purchased from the Model Animal Research Center, Nanjing University, and housed individually in standard cages under controlled conditions (22 ± 2 °C, 50–60% humidity, 12 h light/dark cycle) with ad libitum access to standard chow and water. After a one-week acclimatization period, mice were randomly allocated to five groups (*n* = 10 per group) using a computer-generated randomization sequence (Excel RAND function, block size = 5): (1) Control group—non-infected wounds treated with BC membrane; (2) Infection + BC membrane; (3) Infection + CBC membrane; (4) Infection + BA@BC membrane; (5) Infection + BA@CBC membrane. Inclusion criteria were established a priori: male, SPF, healthy, normal body weight; no animals were excluded. Blinding was maintained throughout: animal care technicians and outcome assessors were blinded to group allocation using coded animal IDs, and the statistician was blinded until analysis completion.

Under intraperitoneal anesthesia with chloral hydrate (0.5 mg/kg), a standardized 10-mm-diameter full-thickness excisional wound was created on the dorsal skin. Four groups were inoculated with 100 μL ATCC 25923 (1 × 10^6^ CFU/mL) to establish infection; the control group received sterile saline. Wound healing progression was monitored and quantified by digital planimetry on post-wounding days 2, 4, 6, and 8. The healing rate is calculated according to the following formula.

(4) $$\mathrm{RC}\;(\%)=\frac{{A_{0}}^{2}-A^{2}}{{A_{0}}^{2}}\times 100\%$$

*A*_0_ is the initial wound width, and *A* is the wound width on the dressing-change day.

On Day 4 and 8, mice were humanely euthanized. Skin tissue specimens, including underlying granulation tissue, were collected, immediately immersed in 4% (*w*/*v*) neutral-buffered paraformaldehyde for 24 h at 4 °C, then processed through a graded alcohols series for dehydration cleared in xylene, and embedded in paraffin wax. Serial 5-μm-thick sections were prepared and subjected to multiple histological and molecular analyses: hematoxylin and eosin (H&E) staining for general morphology and inflammatory cell infiltration, as well as immunohistochemistry (IHC) using primary antibodies against tumor necrosis factor-α (TNF-α) and Interleukin-1β (IL-1β). The expression of TNF-αand IL-1β was analyzed and quantitatively scored using Image Pro Plus 6.0 software.

### 2.10. Statistical Analysis

Each experiment was performed in quintuplicate to ensure data robustness and reproducibility. Statistical analyses were conducted using SPSS software (version17.0). Continuous variables are expressed as mean ± standard deviation (SD). Multiple sample means were compared by one-way ANOVA, followed by pairwise comparisons with t-test. Statistically significant differences were denoted when *p* < 0.05.

## 3. Results and Discussion

### 3.1. Construction and Characterization of the BA@Col/BC Dressing

**Figure 1:**
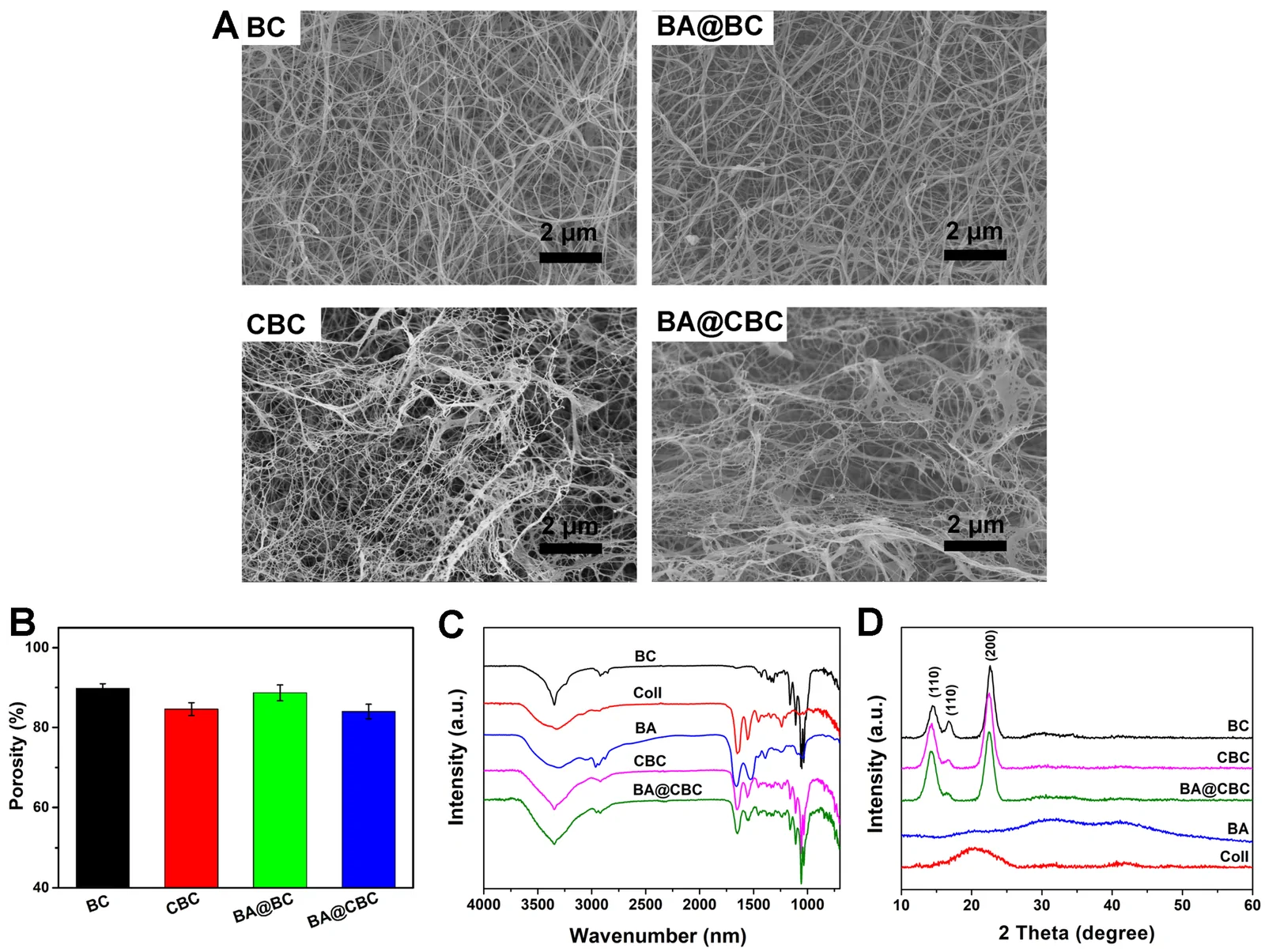
Characterization of BA@Col/BC dressing. (**A**) SEM; (**B**) porosity; (**C**) FTIR spectra; (**D**) XRD patterns.

The surface morphologies of BC, CBC, BA@BC and BA@CBC were observed by SEM. As shown in Figure 1A, the BC membrane has a typical 3D porous nanofiber network structure with smooth fiber surfaces, which is consistent with the results reported in the literature [22]. The surface morphology and fiber state of BA@BC show no significant difference from those of BC, possibly because BA is a small molecule and adheres to the surface of BC nanofibers. In the network structures of CBC and BA@CBC, a layer of collagen-formed membrane can be clearly observed, but the nanoporous network structure is still retained. From the porosity test results, it can be seen that the porosity of BC and BA@BC is as high as 90%, while that of CBC and BA@CBC slightly decreases to 86% and 85%, respectively (Figure 1B). The high porosity is conducive to the rapid removal of exudate [23].

In this experiment, ATR-FTIR was used to characterize the chemical composition of the materials. As shown in Figure 1C, BC exhibited absorption peaks at 3345 cm^−1^ and 2900 cm^−1^, which were attributed to the stretching vibration of the O-H bond and the asymmetric stretching vibration of the C-H bond on the cellulose chain, respectively [24,25]. For ColI, absorption peaks were observed in the range of 3400–3100 cm^−1^, corresponding to the stretching vibration of the N-H bond. Additionally, Col presented absorption peaks at 1650 cm^−1^ and 1545 cm^−1^, which were attributed to the characteristic absorption of the amide I band (C=O) and the amide II band (N-H), respectively [26]. In the infrared spectrum of BA, distinct absorption peaks of the amide I band and the amide II band were visible [27]. Compared with BC, the Col/BC composite (CBC) showed significant infrared absorption peaks at 1650 cm^−1^ and 1545 cm^−1^, corresponding to the characteristic absorption of the amide I band (C=O) and the amide II band (N-H), respectively, indicating that Col had successfully been combined with BC. Similarly, BA@BC also presented obvious absorption peaks of the amide I band and the amide II band at 1650 cm^−1^ and 1545 cm^−1^, confirming the effective combination of BA and BC. For the ternary composite BA@CBC, compared with CBC or BA@BC, no new characteristic absorption peaks were observed due to the peak overlap of the characteristic absorption peaks of BA and Col in the composite material. However, the absorption intensities of the amide I band and the amide II band were higher than those of CBC and BA@BC after double loading, indicating that BA and Col had been co-loaded onto the BC substrate.

To investigate whether the introduction of BA and Col would affect the crystal structure of BC, XRD tests were conducted on the samples. As shown in Figure 1D, BC presented three diffraction peaks at 14.8°, 16.5° and 22.7°, corresponding to the (110), (110) and (200) diffraction planes of BC [28]. For Col, a broad peak appeared around 15–30°, which was consistent with the results reported in the literature [29]. Since no diffraction peaks were observed in the XRD pattern of BA, it indicated that the structure of BA was mainly amorphous. The XRD patterns of CBC, BA@BC and BA@CBC were consistent with the peak positions of BC, suggesting that the introduction of Col or BA did not disrupt the crystal structure of BC.

### 3.2. Thermostability of the BA@CBC Dressing

**Figure 2:**
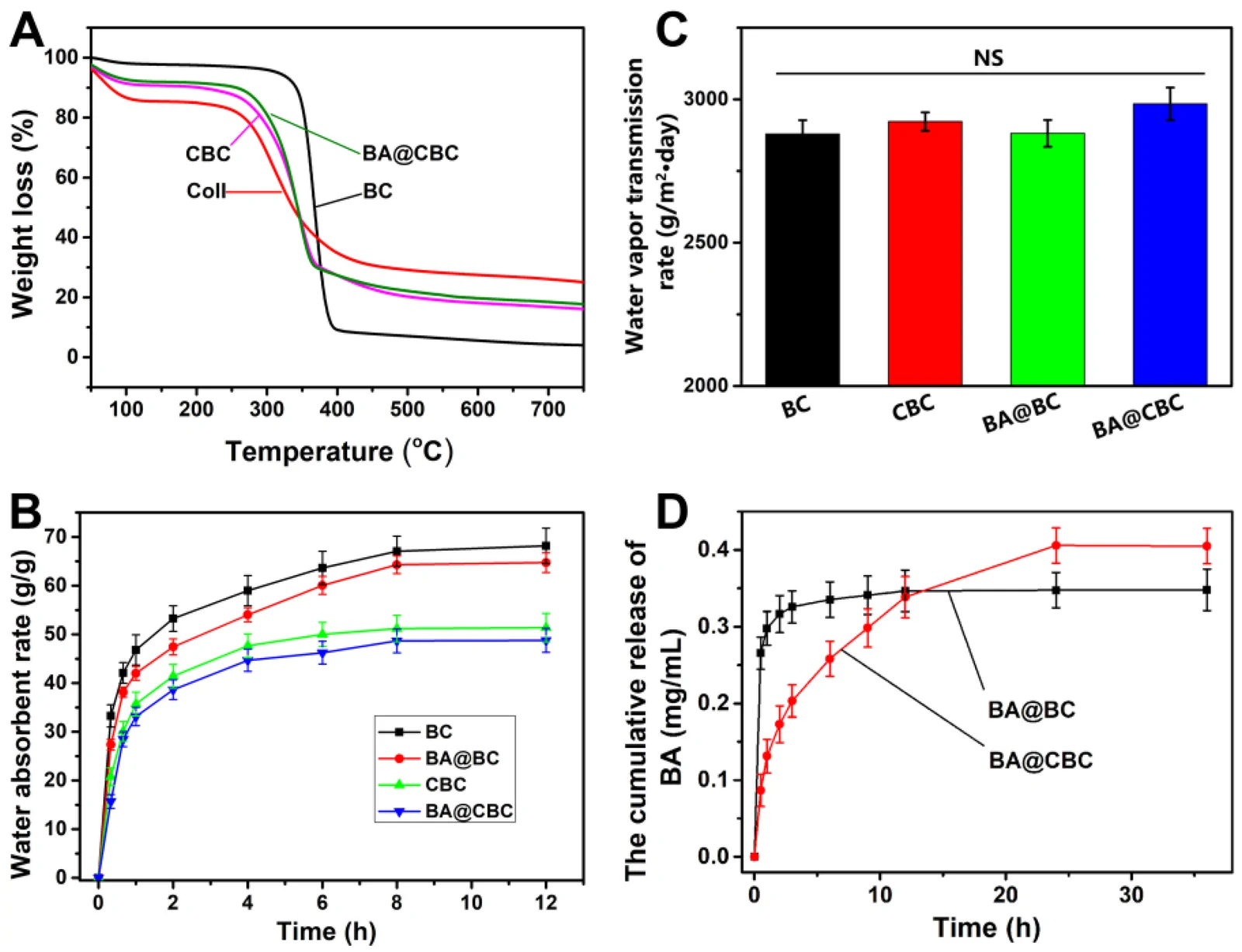
Physicochemical properties of BA@CBC dressing: typical thermal degradation profiles (**A**), water absorption curves (**B**), water vapor transmission rates (**C**) and the cumulative release curve of BA (**D**). (NS: no significant difference)

Figure 2A shows the TG curves of the samples. As can be seen from the figure, all samples exhibit two temperature intervals of mass loss. The first interval (room temperature–100 °C) is attributed to the evaporation of adsorbed free water in the samples. BC has a mass loss of approximately 5%, while CBC, BA, BA@BC, and BA@CBC all have mass losses of less than 10%. In contrast, Col shows a mass loss of 15%, which is due to its excellent hydrophilicity, making it prone to adsorbing water vapor from the air. The second mass loss interval for BC occurs at 300–400 °C, with a mass loss as high as 95%, resulting from the depolymerization of cellulose molecules and the decomposition of glucose rings [30]. After the introduction of BA and Col, the onset temperature of mass loss for BA@CBC shifts earlier to 280 °C, yet with a mass loss of 81%, it still demonstrates good thermal stability.

### 3.3. Water Absorbency and Water Vapor Transmission Rate

Figure 2B shows the water absorption curves of the samples. All samples exhibited rapid water absorption, reaching over 75% of their saturated water absorption capacity within 2 h. This is attributed to the fact that the obtained dressings retained the nanoporous structure of BC, with porosities all exceeding 85%. On the other hand, BC exhibited a high water absorption ratio of up to 68 g/g, and the introduction of BA had little effect on this ratio. In contrast, after the incorporation of Col, the water absorption ratios of CBC and BA@CBC decreased to 49 g/g and 47 g/g, respectively, which are still significantly higher than the clinical requirement for water absorption performance (>20 g/g). The rapid water absorption capability and relatively high water absorption ratio contribute to the timely removal of exudate by the BA@CBC dressing [31].

The moisture permeability of BC-based dressings is shown in Figure 2C. The moisture permeability of BC dressing reached 2800 g/m^2^ within 24 h, which is significantly higher than the clinical requirement of 1500 g/m^2^ [32]. After the introduction of BA and Col, the moisture permeability of BC-based dressings showed no significant change, owing to the retained nanoporous network structure and high porosity; the moisture permeability of BA@CBC was 2900 g/m^2^. The high moisture permeability ensures effective water vapor transmission, reduces the risk of moisture accumulation, and maintains a moist healing environment.

### 3.4. The Release Kinetics of BA

Figure 2D presents the cumulative release profiles of BA. BA@BC exhibited a pronounced burst release characteristic, with over 90% of the drug released within 4 h and complete release achieved by 12 h. In contrast, BA@CBC demonstrated favorable sustained-release behavior, maintaining continuous drug release for up to 24 h. Furthermore, the cumulative release concentration of BA from BA@CBC (0.4 mg/mL) was significantly higher than that from BA@BC (0.34 mg/mL), indicating that the incorporation of Col not only effectively retarded the release rate of BA but also substantially enhanced the drug loading capacity. The observed differences can be primarily attributed to the distinct drug loading mechanisms of BC and Col. Although BC possesses a nanoporous network structure with hydroxyl-rich fiber surfaces, its relatively large pore size offers limited physical constraint for small-molecule BA, resulting in rapid drug diffusion and loss, insufficient binding sites, and consequently low drug loading. In comparison, Col exhibits excellent drug-loading properties: the molecular chains of Col contain abundant active functional groups (such as amino and carboxyl groups), which can interact with drug molecules through physical adsorption or chemical bonding. When Col attaches to the surface of BC fibers or fills the interstitial spaces of the BC network, it not only increases the number of drug binding sites and enhances loading capacity, but also forms a denser diffusion barrier, thereby achieving a sustained-release effect. Numerous studies have demonstrated that type I collagen exhibits excellent drug-loading capacity and sustained-release properties [33,34,35]. In this study, bacitracin was successfully loaded into and sustainedly released from type I collagen; however, the drug utilization efficiency still warrants further optimization. In the future, we intend to employ pH-responsive dynamic crosslinking to immobilize bacitracin, aiming to achieve spatiotemporally precise regulation of drug release at infected wound sites, which will constitute the focus of our next-stage exploration.

### 3.5. Antibacterial Activities of the BA@CBC Dressing

**Figure 3:**
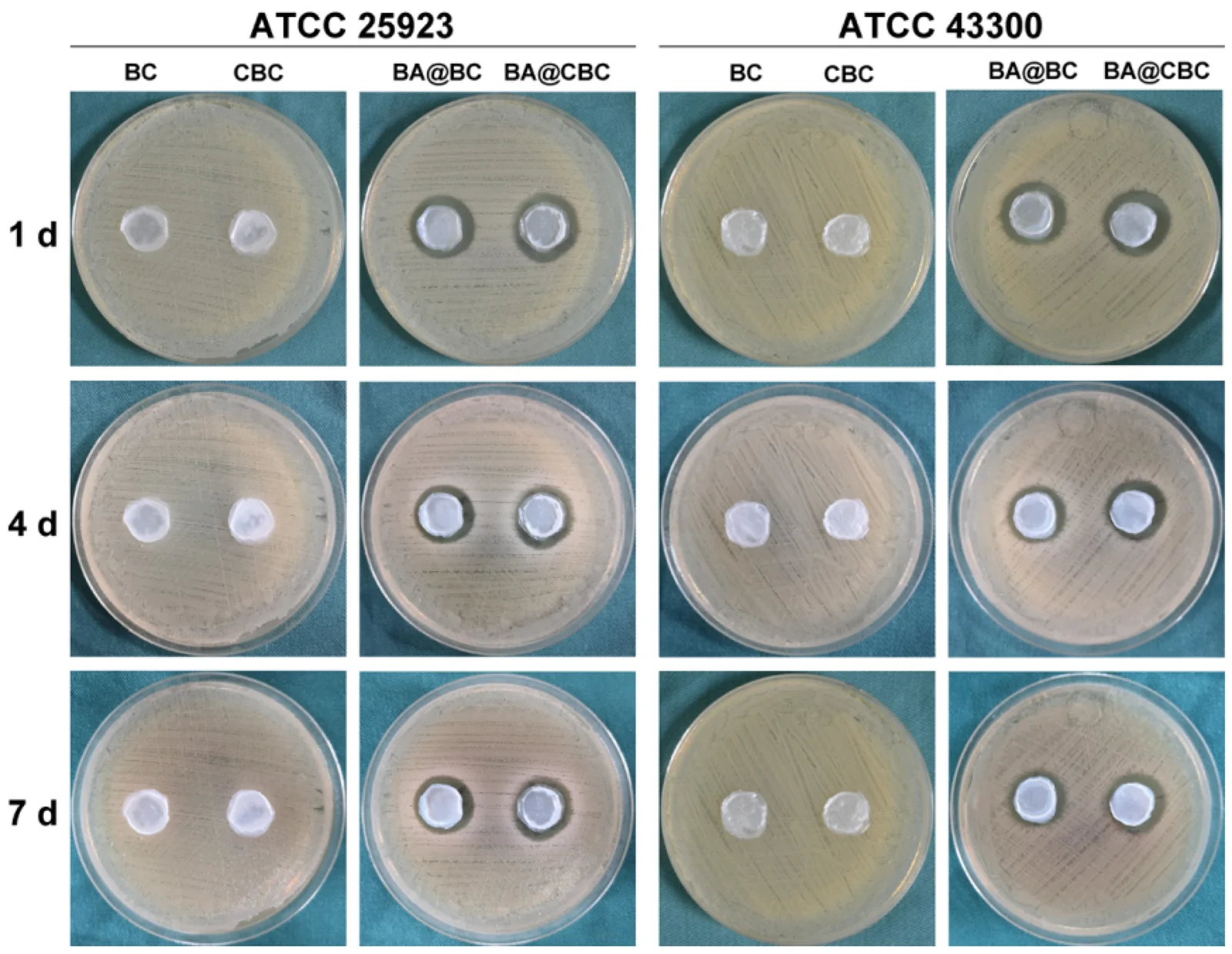
The ZOI against *S. aureus* and *MRSA* after incubation for 1, 4, and 7 d.

Figure 3 presents photographs of the inhibition zones for each group. As BC and Col inherently lack antibacterial activity, no inhibition zones were observed in either the BC or CBC group. In contrast, both BA-loaded samples (BA@BC and BA@CBC) exhibited distinct inhibition zones against the two pathogenic bacteria. Furthermore, the antibacterial efficacy of both BA-loaded groups persisted through day 7, indicating that the incorporation of BA successfully conferred excellent prolonged antibacterial properties upon the BC-based dressings. Importantly, the BA@CBC sample displayed markedly bigger antibacterial inhibition zones compared with BA@BC. Such a difference verifies that collagen introduction improves BA loading capacity and facilitates its sustained release, which collectively strengthens the antibacterial property of BA@CBC.

**Figure 4:**
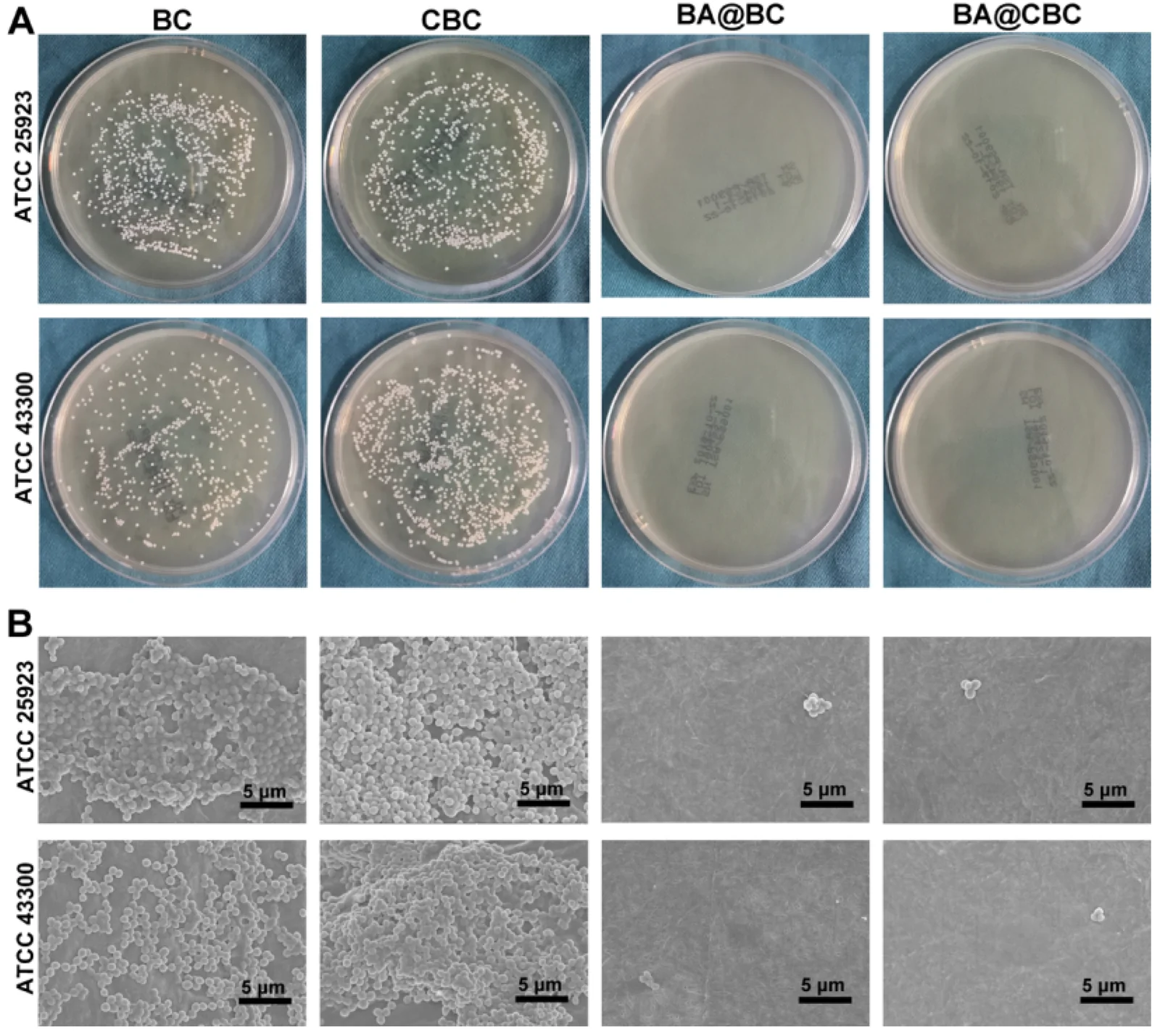
Antibacterial activities of BA@CBC. (**A**) Viable bacteria on sample surfaces determined by the spread plate method; (**B**) SEM images of colonized bacteria.

To further verify the antibacterial efficacy of BA@CBC, a quantitative evaluation was conducted using the spread plate counting method. As shown in Figure 4A, the agar plates of both the BC and CBC groups were densely covered with bacterial colonies of the two strains, whereas no bacterial colonies were detected on those of the BA@BC and BA@CBC groups. Based on these results, the antibacterial rates of both BA-loaded samples against the two bacteria were calculated to exceed 99%. This finding was further corroborated by SEM observations: both bacterial strains formed thick biofilms on the surfaces of BC and CBC, while only sporadically scattered bacteria were observed on the surfaces of BA@BC and BA@CBC (Figure 4B). The excellent antibacterial performance of BA has also been documented in the literature [36,37,38].

### 3.6. Cytocompatibility of the BA@CBC Dressing

**Figure 5:**
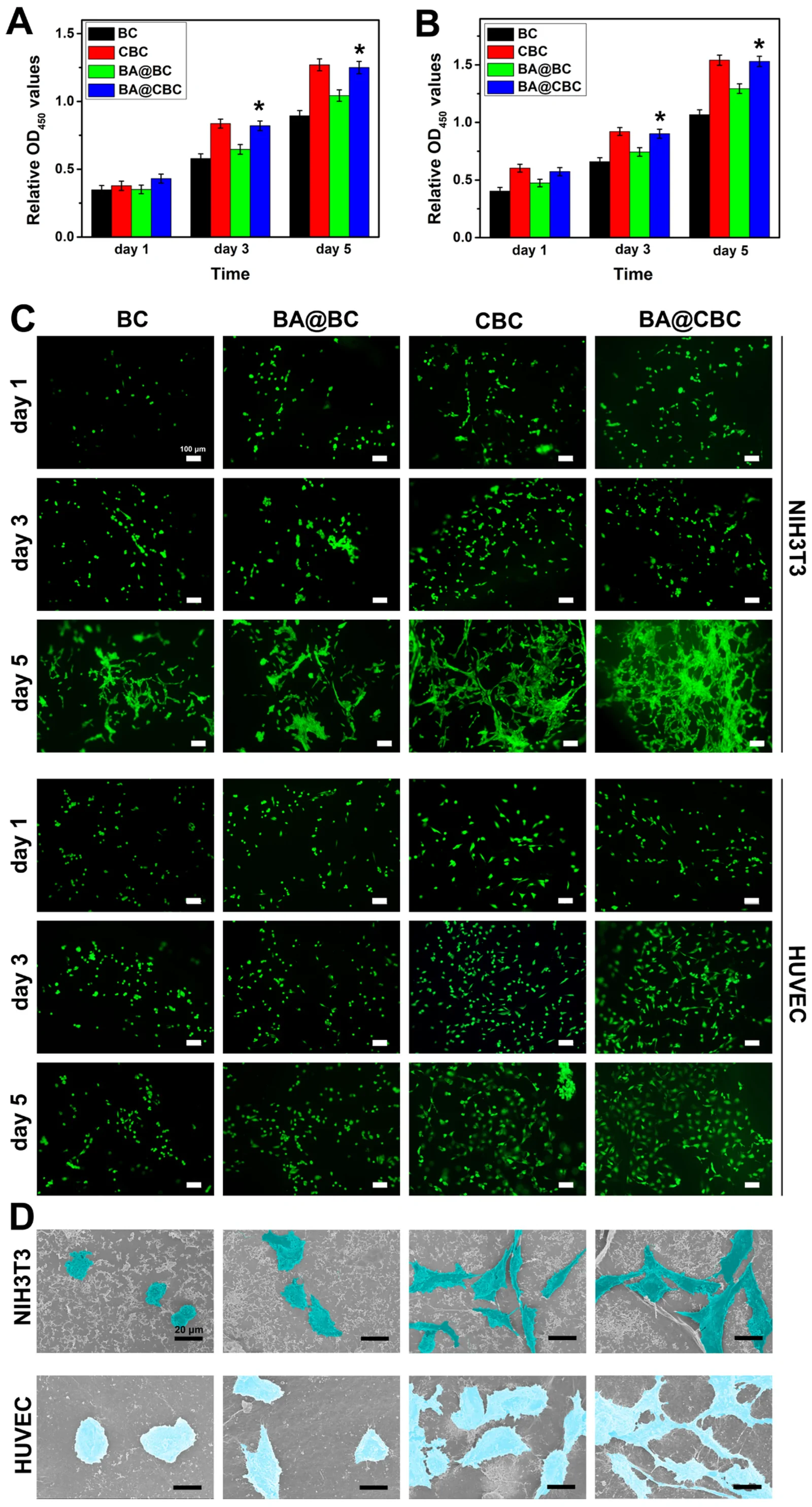
Cytocompatibility of the BA@CBC dressing. (**A**) Proliferation of NIH3T3 by CCK-8 assay; (**B**) Proliferation of HUVECs by CCK-8 assay; (**C**) live & dead cell staining. * *p* < 0.05 compared BA@CBC with BC and BA@BC; (**D**) SEM images of cells on samples.

The proliferation of NIH3T3 (Figure 5A) and HUVECs (Figure 5B) on material surfaces was evaluated using the CCK-8 assay. The results demonstrated that the incorporation of both BA and Col promoted cell proliferation without observable cytotoxicity. Although BA possesses antibacterial activity, it could still facilitate cell growth at an appropriate dosage, which is consistent with our previous findings [11,13]. Notably, Col exhibited a significantly stronger promoting effect on cell proliferation than BA, reflecting its excellent cytocompatibility. In addition, no statistically significant difference was observed between the CBC and BA@CBC groups. These conclusions were further corroborated by live/dead cell staining. As shown in Figure 5C, no red fluorescence (dead cells) was detected in any of the samples, confirming the absence of cytotoxicity across all groups. Moreover, the cell number increased over time in all groups, with the BA@BC group showing a higher cell density than the BC group, the CBC group exhibiting the highest cell number, and the BA@CBC group being comparable to the CBC group.

SEM observation of cell morphology is presented in Figure 5D. Although BC possesses favorable biocompatibility, its cytocompatibility was relatively poor, with cells remaining round and failing to spread on its surface. In contrast, cells on the BA@BC surface showed better spreading than those on BC, while the CBC and BA@CBC groups exhibited the most favorable cell spreading, with cells predominantly displaying spindle-shaped and polygonal morphologies.

### 3.7. In Vivo Wound Healing

**Figure 6:**
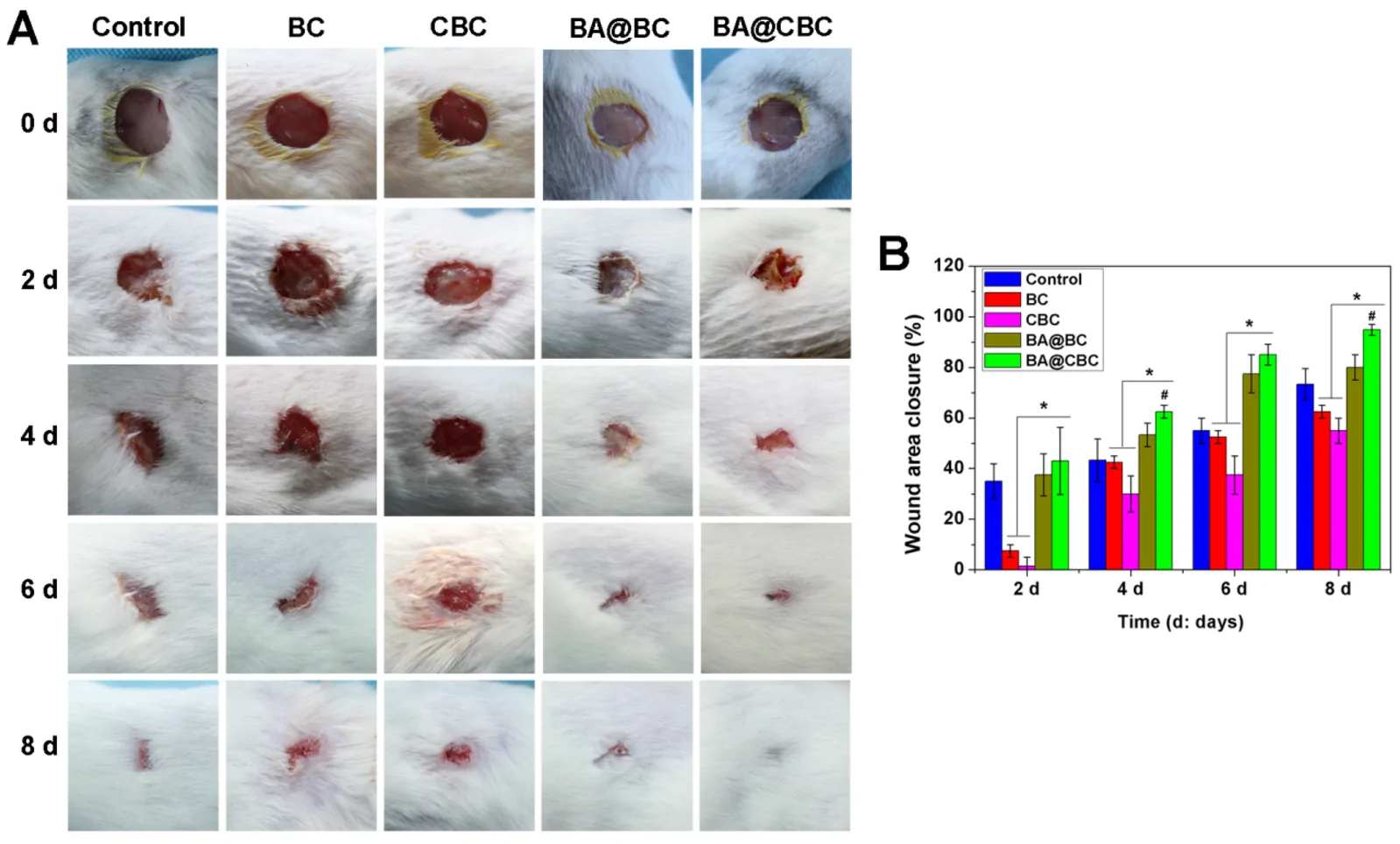
Infected wound care competency assessment for BA@CBC. (**A**) Representative photographs of the wounds on Day 0, 2, 4, 6 and 8 after covering with dressing. The wounds without infection and treated with BC were noted in the control group; (**B**) Wound area closure percentage of wounds, * *p* < 0.05 compared BA@CBC with BC and CBC, # *p* < 0.05 compared BA@CBC with BA@BC.

A full-thickness dorsal skin wound infection model in mice was established to evaluate the therapeutic efficacy of BA@CBC dressings on infected wounds. The macroscopic observation of wound healing is presented in Figure 6A, where the Control group represents uninfected wounds treated with BC dressings. The results demonstrated that infection significantly delayed the wound healing process, with the infected BC group exhibiting markedly inferior wound closure compared to the Control group. Upon the introduction of BA, the BA@BC group showed improved wound healing relative to the BC group, indicating effective suppression of infection. Notably, the CBC group exhibited the poorest wound healing performance. It is postulated that in the absence of effective infection control, Col may paradoxically promote bacterial proliferation, thereby exacerbating infection and further impeding wound healing. Our previous study has demonstrated that in colony counting assays, the Col-composited CBC group exhibited higher bacterial counts than the BC group, indicating that Col promotes bacterial proliferation [39]. Only under the premise of infection eradication by BA can Col fully exert its wound-healing promoting effects. Consequently, the BA@CBC group demonstrated the most favorable pro-healing outcome, achieving complete wound closure within 8 days. It is worth noting that the healing effect of the BA@CBC group was also significantly better than that of the BA@BC group. Statistical analysis revealed that the healing rate of the BA@CBC group was significantly higher than that of the other groups, including BA@BC group, with statistically significant differences observed between BA@CBC and both the BC and CBC groups (Figure 6B). Collectively, this result is attributed to the combined synergistic effects originating from three components: the intrinsic antibacterial performance of BA, tunable drug loading and release characteristics, and the favorable cytocompatibility provided by collagen.

### 3.8. Histological and Immunohistochemical Analyses

**Figure 7:**
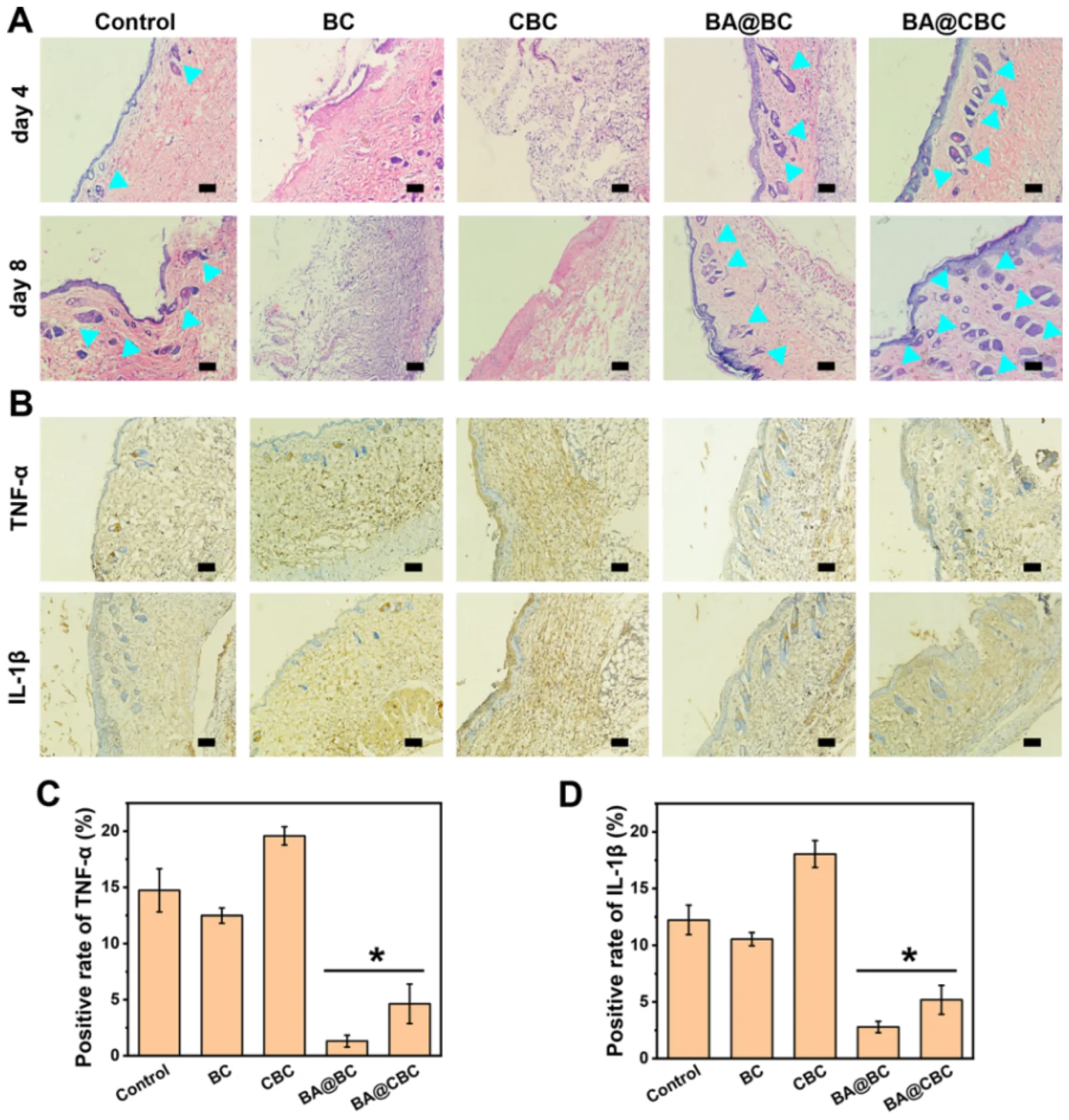
Histological and immunohistochemistry analyses of wounds. (**A**) H&E staining, the blue arrow indicates new hair follicles; (**B**) Immunohistochemical staining for TNF-α and IL-1β at postoperative Day 4; (**C**) Quantitative analysis of TNF-α; (**D**) Quantitative analysis of IL-1β. * *p* < 0.05 compared BA@BC and BA@CBC groups with the other groups. Bar: 100 μm.

H&E staining was performed on wound tissues harvested at postoperative Day 4 and Day 8, with representative images shown in Figure 7A. At Day 4, prominent infiltration and aggregation of macrophages or other mononuclear cells were observed in the wound tissues of the BC and CBC groups. In contrast, the damaged areas in the BA@BC and BA@CBC groups showed markedly reduced inflammatory cell infiltration, attributable to the suppressive effect of BA on macrophage activity. This finding indicates that the incorporation of BA effectively attenuates the local inflammatory response at the wound site. Concurrently, epithelialization structures began to emerge in the wound tissues of both the BA@BC and BA@CBC groups. By Day 8, the BA@CBC group exhibited more regularly organized epithelial and dermal layers compared with the other groups, accompanied by de novo hair follicle formation. These results demonstrate that the BA@CBC group achieved the optimal healing outcome at Day 8, which is consistent with the macroscopic observations described above.

In the wound healing process, inflammation serves as a critical indicator that reflects the infection status of the wound. Therefore, the expression levels of IL-1β and TNF-α were analyzed by immunohistochemistry at postoperative Day 4 (Figure 7B). Marked expression of TNF-α and IL-1β was observed in the CBC group, indicating a significant inflammatory response at the wound site in mice treated with CBC composite materials. In contrast, the expression levels of TNF-α and IL-1β were notably reduced in the other groups, suggesting that the incorporation of Col exacerbates the inflammatory response at the wound site. For the BA@BC and BA@CBC composites supplemented with BA, TNF-α and IL-1β expression was virtually absent. Quantitative statistical analysis clearly revealed that the two BA-loaded groups (BA@BC and BA@CBC) secreted significantly lower levels of TNF-α and IL-1β compared to the other three groups (Figure 7C,D). This can be attributed to the ability of BA to suppress macrophage activity, coupled with its inherent antibacterial properties, which effectively protect the wound from bacterial infection and substantially diminish the local inflammatory response. In our previous studies, BA-modified Ti-based bone implants demonstrated favorable antibacterial, anti-inflammatory, and pro-stem cell proliferation and differentiation properties, as well as the ability to promote healing of infected bone defects [11,12]. In the present study, although the composite dressings exhibited superior efficacy in attenuating inflammation and accelerating wound healing compared to the control groups, the specific contributions of bacitracin versus the physicochemical properties of the dressing matrix remain to be further elucidated. Future investigations will focus on single-component experimental studies to quantitatively assess these independent effects.

## 4. Conclusions

In summary, the bacitracin-loaded type I collagen/bacterial cellulose (BA@CBC) functional dressing was prepared via a two-step method, namely in situ compositing combined with immersion loading. The resulting BA@CBC functional dressing exhibited high porosity, favorable thermal stability, rapid water absorption capacity, and excellent moisture permeability. The incorporation of collagen not only significantly enhanced the drug loading of bacitracin (BA), but also effectively retarded its release rate. The synergistic effect of BA and collagen endowed the dressing with outstanding antibacterial activity, while simultaneously promoting the adhesion, spreading, and proliferation of NIH3T3 and HUVECs. In vivo experimental results further confirmed that the BA@CBC dressing could effectively eliminate wound infection, alleviate local inflammatory responses, and promote granulation tissue formation and hair follicle neogenesis, thereby accelerating the wound healing process. With its multifunctionality and facile preparation process, the BA@CBC dressing demonstrates great potential for treating infected wounds.

## Data Availability

The original contributions presented in the study are included in the article, further inquiries can be directed to the corresponding author.
